# Characterizations of distinct parallel and antiparallel G-quadruplexes formed by two-repeat ALS and FTD related GGGGCC sequence

**DOI:** 10.1038/s41598-018-20852-w

**Published:** 2018-02-05

**Authors:** Bo Zhou, Yanyan Geng, Changdong Liu, Haitao Miao, Yaguang Ren, Naining Xu, Xiao Shi, Yingying You, Tunglun Lee, Guang Zhu

**Affiliations:** 1 Division of Life Science, The Hong Kong University of Science and Technology, Clear Water Bay, Kowloon, Hong Kong SAR People’s Republic of China; 2 Institute for Advanced Study, The Hong Kong University of Science and Technology, Clear Water Bay, Kowloon, Hong Kong SAR People’s Republic of China

## Abstract

The large expansion of GGGGCC (G4C2) repeats of the *C9orf72* gene have been found to lead to the pathogenesis of devastating neurological diseases, amyotrophic lateral sclerosis (ALS) and frontotemporal dementia (FTD). The structural polymorphisms of *C9orf72* HRE DNA and RNA may cause aberrant transcription and contribute to the development of ALS and FTD. Here we showed that the two-repeat G4C2 DNA, d(G4C2)_2_, simultaneously formed parallel and antiparallel G-quadruplex conformations in the potassium solution. We separated different folds of d(G4C2)_2_ by anion exchange chromatography, followed with characterizations by circular dichroism and nuclear magnetic resonance spectroscopy. The parallel d(G4C2)_2_ G-quadruplex folded as a symmetric tetramer, while the antiparallel d(G4C2)_2_ adopted the topology of an asymmetric dimer. These folds are distinct from the antiparallel chair-type conformation we previously identified for the d(G4C2)_4_ G-quadruplex. Our findings have demonstrated the conformational heterogeneity of the *C9orf72* HRE DNA, and provided new insights into the d(G4C2)_n_ folding. Meanwhile, the purified d(G4C2)_2_ G-quadruplex samples are suitable for further three-dimensional structure characterizations, which are required for the structure-based design of small molecules targeting ALS and FTD related *C9orf72* HRE.

## Introduction

Amyotrophic lateral sclerosis (ALS) and frontotemporal dementia (FTD) are severe neurological diseases^1–3^. The abnormal expansion of a GGGGCC (G4C2) hexanucleotide repeat (HRE) in a noncoding region of the *C9orf72* gene has been found to be a single genetic cause of ALS and FTD^4–6^. Fewer than 25 G4C2 repeats are present in the healthy control population^7^, whereas more than 700 G4C2 repeats exist in the ALS and FTD patients^4,5^. In patient cells, the G4C2 HRE transcripts accumulate as nuclear RNA foci, which may sequester and inactivate RNA binding proteins^8,9^. The transcribed G4C2 expansion repeats can also undergo non-ATG initiated translation, which result in toxic dipeptide accumulation^10–12^. Despite these findings, the detailed disease mechanism remains to be elucidated. Recent studies have demonstrated that the structure polymorphisms of both *C9orf72* HRE DNA and RNA may contribute to the pathogenesis of ALS and FTD diseases^13^. They can form complex structures, including DNA or RNA G-quadruplexes and DNA∙RNA hybrids, which may cause transcriptional abortion leading to the loss of full-length RNA transcripts and accumulation of abortive RNA transcripts^9,14,15^.

The G-quadruplex is a stable four-stranded structure formed by guanosine-rich DNA and RNA sequences. Hoogsteen hydrogen bonds connect four guanine bases to form a square planar structure called guanine tetrad. Two or more guanine tetrad layers stack to form a G-quadruplex and monovalent cations can stabilize the G-quadruplex formation. Various cations affect the topology and stability of G-quadruplexes differently^16^. G-quadruplexes can adopt parallel, antiparallel, or mixed topology, depending on the orientation of the strand loops^17,18^. In recent years, accumulating evidence point to the existence of G-quadruplex structures under physiological conditions, which may have critical functional roles in many cellular processes. G-quadruplex and other peptides, like β-amyloid, are highly attractive targets for therapeutic interventions for neurological disorders^15,19–21^. G-quadruplex-based assays and probes are also used to detect exonuclease activity or gene deletion^22,23^.

Several studies have investigated *C9orf72* HRE DNA G-quadruplexes^9,24–27^. Circular dichroism (CD) spectroscopy showed that the *C9orf72* HRE DNA with different lengths adopted distinct G-quadruplex topologies. The d(G4C2)G4 formed parallel G-quadruplex; d(G4C2)_4_ formed antiparallel G-quaduplex; d(G4C2)_2_, d(G4C2)_3_ and d(G4C2)_5_ formed mixed G-quadruplexes with mixed topologies^9,25^. Using NMR approaches, we previously solved the topology of d(G4C2)_4_, which exhibited a chair-type G-quadruplex with a four-layer antiparallel G-tetra core and three edgewise loops^25^. In another study, the solution structure of a modified G4C2 DNA, d[(G4C2)_3_GG^Br^GG], was determined to adopted an antiparallel topology with four G-quartets and three edgewise C-C loops^26^.

Previous findings have suggested that the parallel topology is adopted by most *C9orf72* (G4C2)_n_ DNAs^9,24,25^. However, to date, no parallel topology of the *C9orf72* HRE DNA has been determined, largely due to the difficulty imposed by the heterogeneous conformations of G-rich oligonucleotides for structural determination. Commonly, the adjustment of length of oligonucleotide repeats, or mutation and modification of specific nucleotides could be employed to stabilize a particular structural fold of G-quadruplexes. The experimental conditions, including pH, temperature and type of cations in the buffers, could also profoundly impact the G-quadruplex folding.

To obtain a *C9orf72* HRE DNA sample with the parallel topology and of sufficient quality for structural determination, we carefully examined our previous data, including CD and NMR spectra of G4C2 repeats of different lengths^25^. The 1D ^1^H NMR spectra suggested that d(G4C2)_2_ was composed of less heterogeneous G-quadruplex conformations compared to other tested d(G4C2)_n_ DNAs. Therefore, in this study, we employed d(G4C2)_2_ for further purifications, and obtained several distinct fractions by anion exchange chromatography. The native polyacrylamide gel electrophoresis (PAGE) and CD spectroscopy were performed to investigate all the purified fractions, which demonstrated that homogeneous samples each composed of a distinct parallel or antiparallel G-quadruplex fold were obtained. Using NMR approaches, we further characterized the parallel and antiparallel G-quadruplex conformations. The parallel d(G4C2)_2_ G-quadruplex was found to fold as a symmetric tetramer, while the antiparallel d(G4C2)_2_ G-quadruplex adopted the topology of an asymmetric dimer.

## Results

### d(G4C2)_2_ formed mixed parallel and antiparallel topologies

**Figure 1:**
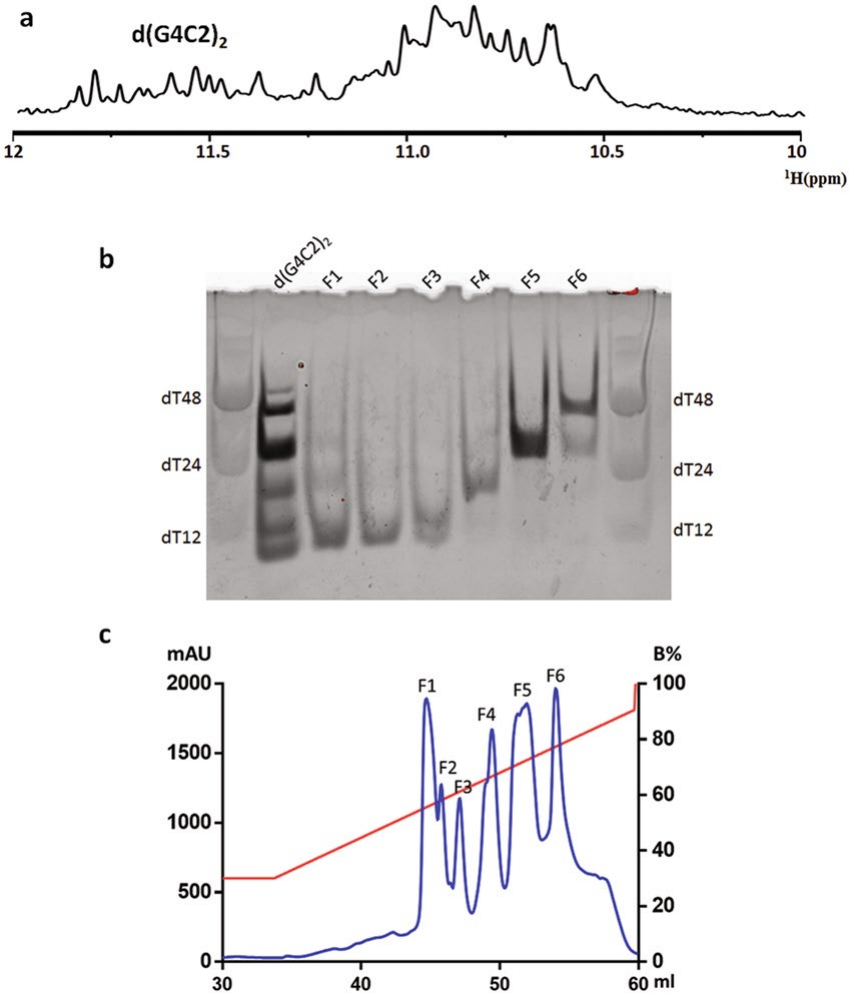
Anion exchange chromatography purification of d(G4C2)_2_. (**a**) The imino region of 1D ^1^H spectrum of d(G4C2)_2_. (**b**) Gel electrophoresis of d(G4C2)_2_ and anion exchange chromatography fractions monitored by the staining method. (**c**) Elution profile of d(G4C2)_2_ from Mono-Q column.

During the preliminary screening of d(G4C2)_n_ samples for structural studies, the CD spectra of d(G4C2)_2_, d(G4C2)_3_ and d(G4C2)_5_ showed a negative peak at 240 nm and positive peaks at 260 and 290 nm^25^. As previous reports presented, characteristic peaks for parallel quadruplexes include a positive maximum at ∼260 nm and a negative minimum at ∼240 nm. Meanwhile, the positive maximum for an antiparallel quadruplex is typically at around 290 nm^28^. Thus, the characteristic peaks of both parallel and antiparallel G-quadruplexes existed in the above CD spectra, indicating mixed topologies in these samples. The NMR spectroscopy was also employed to investigate their folds. The 1D ^1^H spectra of d(G4C2)_n_ except d(G4C2)_2_ showed broaden peaks in the imino region, suggesting the formation of mixtures with multiple G-quadruplex conformations^25^. In contrast, the 1D ^1^H spectrum of d(G4C2)_2_ showed sharp peaks with narrow line widths at 10–12 ppm (Fig. 1a), indicating that its conformation was less heterogeneous. Therefore, we focused on d(G4C2)_2_ in further efforts to obtain a G4C2 DNA G-quadruplex sample with the parallel topology.

To analyze the molecular size of d(G4C2)_2_, the native PAGE was performed and DNA oligonucleotides dT12, dT24 and dT48 were employed as size indicators (Fig. 1b; Supplementary Fig. S1**)**. The d(G4C2)_2_ DNA migrated as multiple bands, in consistency with a mixture of topologies observed in CD spectroscopy.

### Purification of d(G4C2)_2_ by anion exchange chromatography

To separate different conformations of d(G4C2)_2_ in the original sample, anion exchange chromatography was performed using a high resolution Mono-Q column. We generated a potassium chloride gradient to elute DNA fractions from the column using buffer A (20 mM Tris, 50 mM KCl, pH7) and buffer B (20 mM Tris, 1 M KCl, pH7). Twenty-five column volumes of potassium chloride concentration from 30% B to 90% B produced six distinct peaks during the elution process (Fig. 1c). Native PAGE demonstrated that each fraction contained particular components of d(G4C2)_2_ G-quadruplex folds (Fig. 1b). The d(G4C2)_2_ component with lower stoichiometry was eluted at lower salt concentrations. The elution peaks of fractions 1 and 2 (F1 and F2) were closely adjacent and each contained a single band that migrated faster than dT12. Since the monomeric d(G4C2)_2_ could not form a G-quadruplex, the result suggested that F1 and F2 were consisted of homogeneous dimeric G-quadruplexes of similar conformations. Several bands were observed in F3 or F4, which migrated between dT12 and dT24, indicating the composition of different low-order G-quadruplexes. F5 migrated as a single band near dT24, which was likely a homogeneous tetrameric G-quadruplex. In F6, several bands migrated slower than dT24, indicating the composition of high-order structural forms. To further validate the oligomeric status, we compared the electrophoretic mobility of F1 and F5 with monomeric 23-nt human telomere h-telo d[TAGGG(TTAGGG)_3_]^29^ and dimeric 16-nt 93del d[GGGGTGGGAGGAGGGT]^30^ (Supplementary Fig. S2). F1 migrated similarly as h-telo, supporting the dimeric form of d(G4C2)_2_. F5 migrated slower than 93del, which agreed with the tetrameric composition of d(G4C2)_2_.

### CD spectroscopy characterizations of purified d(G4C2)2 fractions

**Figure 2:**
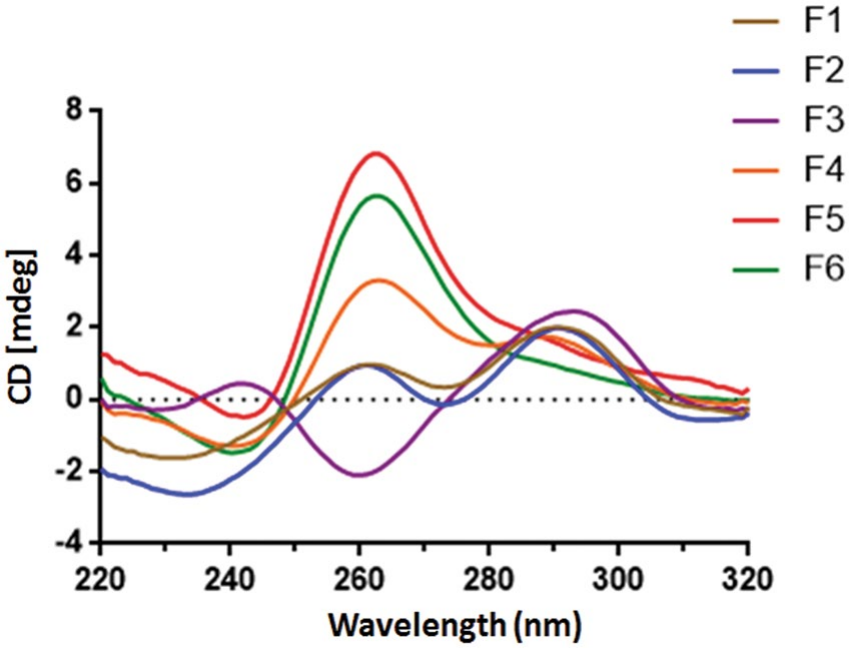
CD spectra of anion exchange chromatography fractions of d(G4C2)_2_. The concentration of each fraction was 20 μM.

To investigate the conformation of d(G4C2)_2_ in different fractions, CD spectroscopy was employed to analyze the topology of each fraction (Fig. 2). In addition to characteristic peaks for parallel and antiparallel quadruplexes as described above, the 290-nm peak indicates that two adjacent G-tetrads have opposite hydrogen-bond directionalities, whereas the 260-nm peak is characteristic of a G-quadruplex in which all the G-tetrads have the same polarity^31^. F1 and F2 fractions displayed two positive maxima at around 260 and 290 nm, and a negative minimum at around 240 nm. Therefore, based on their CD spectra, F1 and F2 adopted a mixed parallel/antiparallel topology with G-tetrad neighbors of mixed relative polarities.

In contrast, F3 displayed a single positive absorption peak and a negative peak at 290 nm and 260 nm, respectively, suggesting the formation of a predominantly antiparallel G-quadruplex fold. The spectrum of F4 exhibited two positive peaks at 260 and 290 nm, which was probably resulted from its mixed conformations as observed in the native PAGE. The CD spectra of F5 and F6 displayed a positive peak at 260 nm and a negative peak at 240 nm, respectively, indicating parallel G-quadruplex folds. These results also demonstrated that the lower stoichiometry of d(G4C2)_2_ was prone to adopt antiparallel topology and the higher stoichiometry of d(G4C2)_2_ favored parallel topologies.

### Stability of d(G4C2)_2_ fractions investigated by CD melting experiments

**Figure 3:**
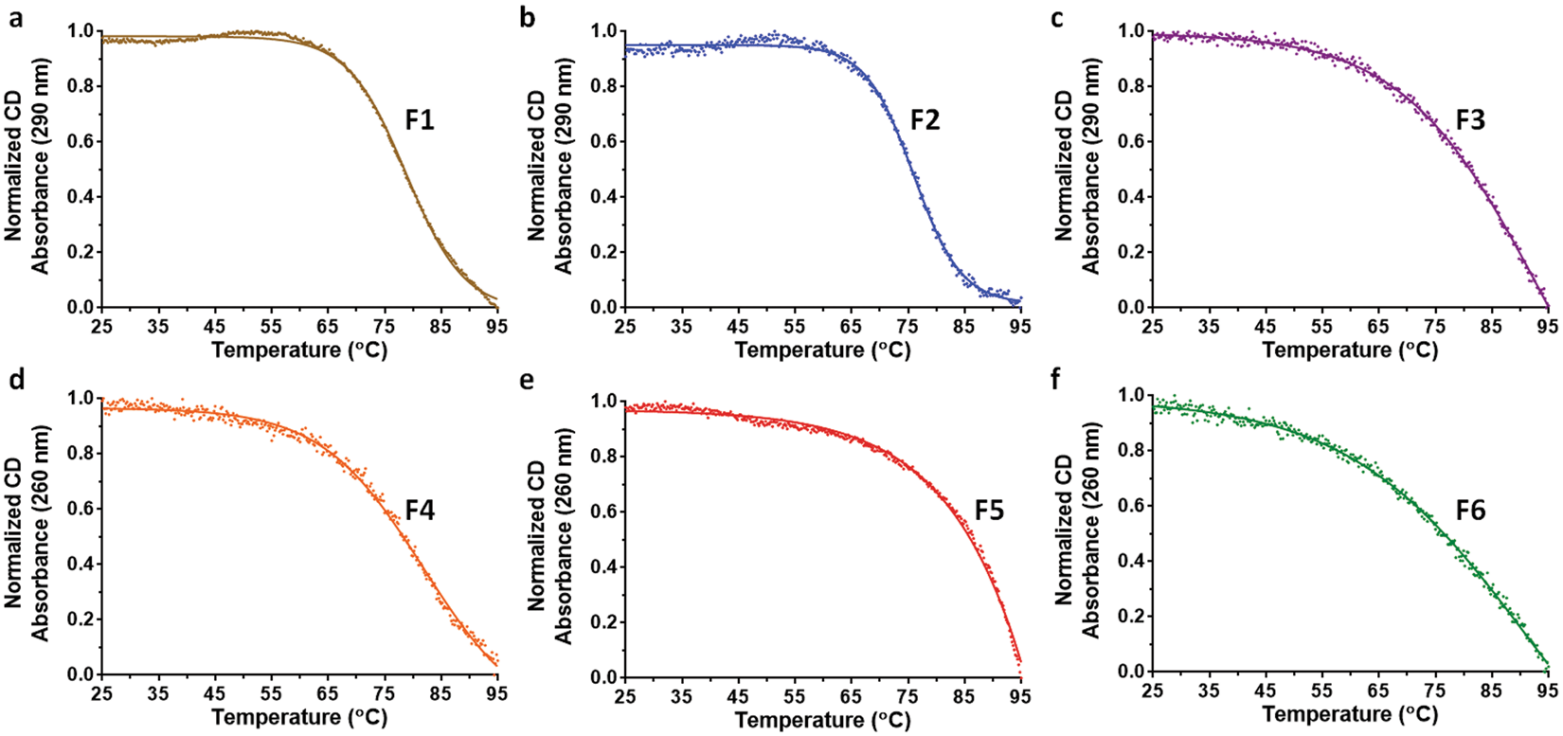
CD melting curves of anion exchange chromatography fractions of d(G4C2)_2_. The melting experiments were performed with a temperature range of 25 °C to 95 °C at 1 °C/min. The CD absorbance was measured at a single wavelength (290 nm for F1, F2 and F3, and 260 nm for F4, F5 and F6). Data were fit by the Boltzmann sigmoid equation (Prism).

The thermal stability of the purified d(G4C2)_2_ fractions were examined by CD melting experiments, which were performed with a temperature range of 25 °C to 95 °C at 1 °C/min. The CD absorbance was measured at a single wavelength (290 nm for antiparallel G-quadruplexes F1, F2 and F3, and 260 nm for parallel G-quadruplexes F4, F5 and F6). The normalized CD absorbance was fitted by the Boltzmann sigmoid equation (Fig. 3). The melting temperatures of F1 and F2 were 78.79 °C and 75.96 °C, respectively. Both their melting curves exhibited apparent transitions, suggesting the stable G-quadruplex structure formation by these DNA samples. In contrast, the melting profiles of F3, F4, F5 and F6 showed no obvious transitions, indicating that these DNA samples formed less stable G-quadruplex structures or adopted multiple conformations. As such, the d(G4C2)_2_ G-quadruplexes in F1 and F2 adopted the most stable conformations among the purified samples.

### Detailed G-quadruplex topologies revealed by NMR spectroscopy

**Figure 4:**
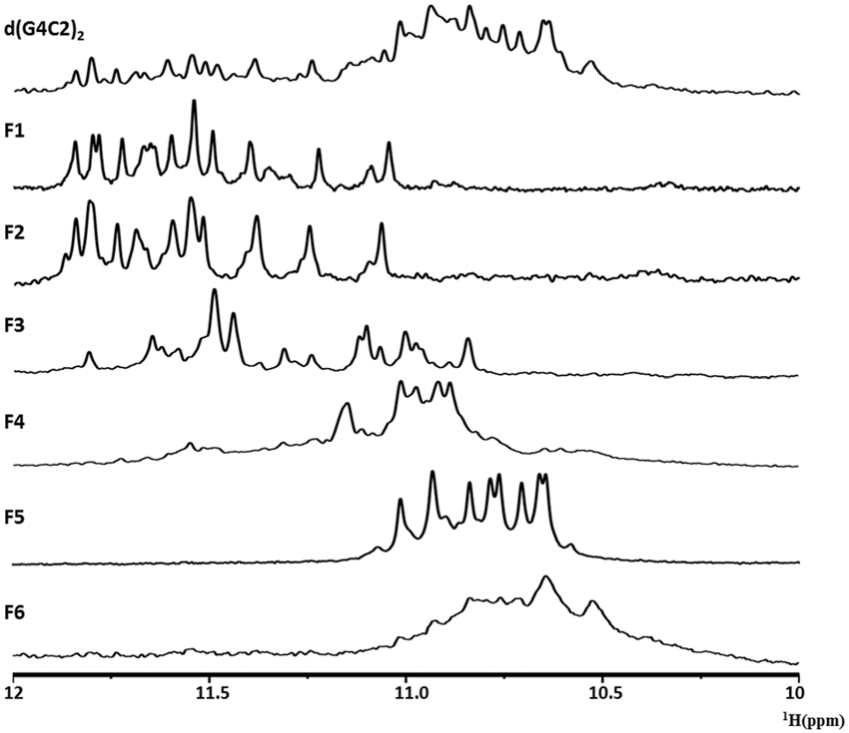
1D ^1^H spectra showed the imino region of d(G4C2)_2_ and anion exchange chromatography fractions. The spectra were recorded in 90% H_2_O, 10% D_2_O at 25 °C.

To obtain more structural insights into d(G4C2)_2_ G-quadruplex conformations, 1D ^1^H NMR spectra were examined for the unpurified d(G4C2)_2_ and the purified d(G4C2)_2_ fractions (Fig. 4). The imino region of ^1^H NMR spectrum of unpurified d(G4C2)_2_ exhibited signals between 10 and 12 ppm, corresponding to imino protons of guanines involved in G-tetrads. Interestingly, the imino region of the antiparallel-dominant G-quadruplex fractions (F1 and F2) showed peaks between 11 and 12 ppm, while that of the parallel-dominant G-quadruplex fractions (F5 and F6) lay between 10.5 and 11 ppm. Thus, the location of the imino peaks may be indicative of the topology of d(G4C2)_n_ G-quadruplexes. The ^1^H NMR spectra of F3 and F4 contained peaks in both antiparallel and parallel regions, indicating the formation of heterogeneous mixtures. The spectrum of F6 showed broaden peaks, which was consistent with the high-order structures determined by the native PAGE experiment.

In 1D ^1^H NMR spectra of F1 and F2, at least 12 imino proton resonances were observed at 10–12 ppm with narrow line widths, indicating the formation of an asymmetric dimeric G-quadruplex fold with three or four G-tetrads. The hydrogen-deuterium exchange (HDX) experiment showed that many imino protons were exposed and subjected to exchange with solvent after 30 min in D_2_O (Supplementary Fig. S3a,b), in consistency with a dimeric G-quadruplex conformation. Meanwhile, the 1D ^1^H NMR spectrum of F5 exhibited 8 well-resolved peaks at 10–12 ppm, indicating a symmetric tetrameric conformation with eight G-tetrads. Around six peaks were well-protected from the exchange with solvent in the HDX experiment, which was consistent with the G-quadruplex fold containing eight tetrad planes (Supplementary Fig. S3c).

No obvious signals were detected in the Watson-Crick base pairing region, implicating that the cytosines were located in the loop region of the d(G4C2)_2_ conformation. The detailed guanine compositions and loop orientations of the asymmetric dimeric and symmetric tetrameric G-quadruplexes remain unclear and need further experiments to resolve.

### Purified d(G4C2)_2_ G-quadruplex fractions are suitable for investigations by crystallography

To evaluate the sample quality of these anion exchange fractions for further structure determination, we employed the X-ray crystallography approach. We screened various crystallization conditions for fractions F1 and F5. In initial trials we obtained crystals of fraction F5 (Supplementary Fig. S4), and a diffraction data set was collected to 1.8 Å resolution. This suggests that the purified samples are suitable for high resolution structural investigations. Since this set of diffraction data is still not sufficient to determine the F5 structure by the molecular replacement method, we will optimize crystal conditions or use bases with heavy-atom substitutions to solve the phase problem in the future study.

## Discussion

The G-quadruplexes formed by the *C9orf72* (G4C2)_n_ DNA or RNA have been found to lead to the pathogenesis of devastating neurological diseases ALS/FTD. The detailed structure information of these G-quadruplexes is critical for understanding the related disease etiology. Previous studies have demonstrated that the *C9orf72* (G4C2)_n_ DNA formed parallel and/or antiparallel topologies^9,24,25^. The d(G4C2)_4_ formed 100% antiparallel topology, while all the other tested d(G4C2)n contained the parallel G-quadruplex topology. Despite topologies resolved for the antiparallel G-quadruplex folds of d(G4C2)_4_^25,26^, the knowledge of the parallel G-quadruplex conformation adopted by the (G4C2)_n_ HRE DNA was limited to indications from CD spectroscopy^9,24,25^.

The structure polymorphisms of G-rich oligonucleotides create the obstacle for obtaining homogenous parallel G-quadruplex folds for the 3-dimensional structure determination. Some modification tools, such as the 8-bromo guanine substitution favoring syn conformation, have been employed to improve the homogeneity of the samples. However, using these approaches, many mutational combinations need to be tested to find the optimal one, which is labor-intensive, tedious and time consuming. In some cases, no guanine with syn conformation in the G-quadruplex is available for chemical modifications. Therefore, other approaches need to be considered for purification of homogeneous G-quadruplex samples for structural determination.

Although anion exchange chromatography has not been frequently used in the structure study of G-quadruplexes, it represents one of the most common methods for the purification of oligonucleotide samples. It is widely used to purify DNA or RNA before crystallization trials^32^. Here we demonstrated that using the method of anion exchange chromatography, we could isolate homogeneous d(G4C2)_2_ samples with either the parallel or antiparallel G-quadruplex conformation. Therefore, the approach of anion exchange chromatography could be routinely adopted to purify distinct conformations of G-quadruplexes for structural characterizations.

In summary, we presented the first report of the purification and characterization of the parallel topology adopted by the G4C2 repeat DNA. Using the method of anion exchange chromatography, we isolated homogeneous d(G4C2)_2_ samples with either parallel or antiparallel G-quadruplex conformation. Further CD and NMR spectroscopy characterizations suggested that the parallel d(G4C2)_2_ G-quadruplex formed a symmetric tetramer while the antiparallel G-quadruplex folded into an asymmetric dimer. Moreover, the purified d(G4C2)_2_ G-quadruplex fractions provided suitable samples for further 3-dimensional structure determinations, which may build the necessary structural basis for designing small molecules targeting ALS and FTD related C9orf72 HRE.

## Methods

### Sample preparation

DNA oligonucleotides were chemically synthesized by IDT. The synthesized DNA was solved at 100 μM concentration in 20 mM potassium phosphate (pH 7.0) and 70 mM KCl. The solved DNA was heated to 95 °C for 15 min, and slowly cooled to room temperature. The NMR sample was concentrated with the use of a Centricon 3kD ultrafiltration column (Millipore, MA).

### Circular dichroism spectroscopy

Circular dichroism spectra were recorded with 400 μl SNA samples at room temperature on an Applied Photophysics Chirascan CD spectrometer using a 1 mm path length quartz cell. DNA concentration was 20 μM. The DNA samples were prepared in the buffer containing 20 mM potassium phosphate (pH 7.0) and 70 mM KCl.

### CD melting

The CD melting experiments were performed with a temperature range from 25 °C to 95 °C at 1 °C/min. The CD absorbance were measured at a single wavelength and then normalized by using the equation (Abst − min)/(max − min), in which Abst is the absorbance at a given temperature, max is the maximum absorbance at 260 nm (parallel G-quadruplexes) and at 290 nm (antiparallel G-quadruplexes), and min is the minimum value. Data were fit by the Boltzmann sigmoid equation (Prism).

### Polyacrylamide gel electrophoresis (PAGE)

The 18% TBE polyacrylamide gel (acrylamide:bis-acrylamide 29:1) was used to run non-denaturing PAGE. 20 mM KCl was supplemented in both gel and running buffer (0.5× TBE). The annealed samples were prepared at a strand concentration of 100 μM. For the anion exchange chromatography fractions, 5 μl of each fraction was combined and loaded in the gel. Red-safe staining was employed to reveal gel bands. The images were acquired by the Molecular Imager Gel Doc XR system (Bio-Rad).

### NMR spectroscopy

The 1D ^1^H NMR experiments were performed on 750 MHz and 800 MHz Varian spectrometers. Watergate or Jump-and-Return water suppression techniques were employed for samples in water solution. NMRPipe was used to process NMR spetra. For the HDX exchange experiments, the oligonucleotides samples were annealed and lyophilized. The lyophilized oligonucleotides were resuspended in 99% D_2_O before NMR measurements.

### DNA crystallization

DNA crystals of fraction F1 and F5 were grown by vapor diffusion from hanging drops at 289 K using Natrix HT (Hampton research). The diffraction data were collected on beamline BL-17U of the Shanghai Synchrotron Radiation Facility (SSRF)^33^.

## Electronic supplementary material

Supplementary information
