# Supplementary material for: Characterizations of distinct parallel and antiparallel G-quadruplexes formed by two-repeat ALS and FTD related GGGGCC sequence
Source: Sci Rep. 2018 Feb 5;8:2366. doi: 10.1038/s41598-018-20852-w (PMC5799222; doi:10.1038/s41598-018-20852-w)
Supplement: Supplementary file 1 — Supplementary information [file 41598_2018_20852_MOESM1_ESM.docx]

**Supplementary Data For:**

**Characterizations of distinct parallel and antiparallel G-quadruplexes formed by two-repeat ALS and FTD related GGGGCC sequence**

**Bo Zhou^1,2,+,*^, Yanyan Geng^1,+^, Changdong Liu^1,+^, Haitao Miao^1^, Yaguang Ren^1^, Naining Xu^1^, Xiao Shi^1^, Yingying You^1^, Tunglun Lee^1^, Guang Zhu^1,*^**


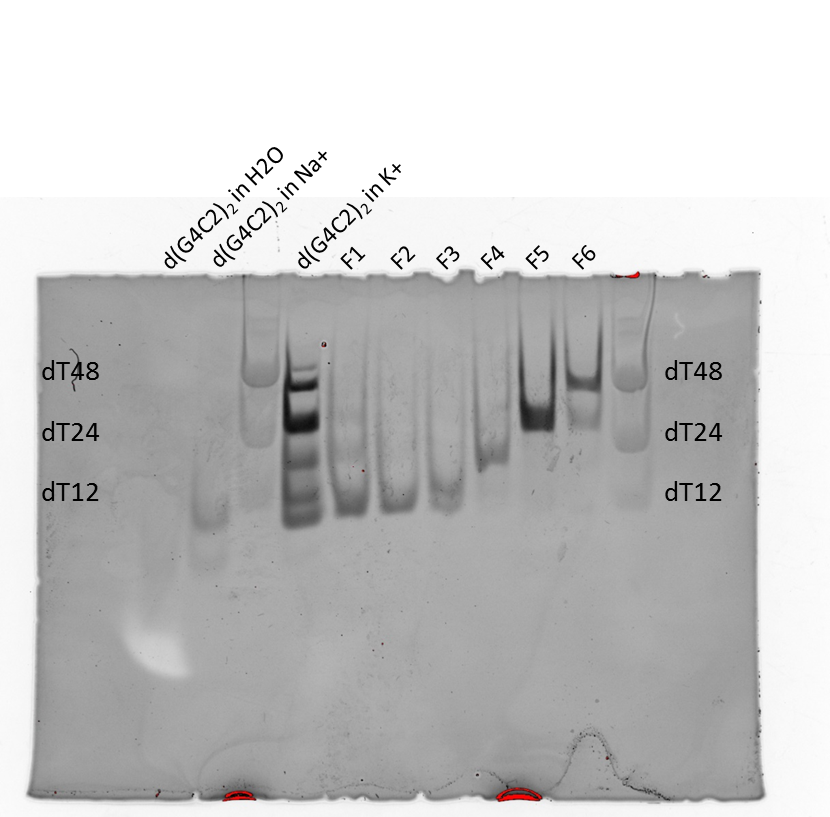


**Figure S1.** **Gel electrophoresis of d(G4C2)_2_ and anion exchange chromatography fractions monitored by the staining method.** This is the original full-length gel figure of Fig. 1b. d(G4C2)_2_ can’t form stable G-quadruplex in H2O or sodium chloride.


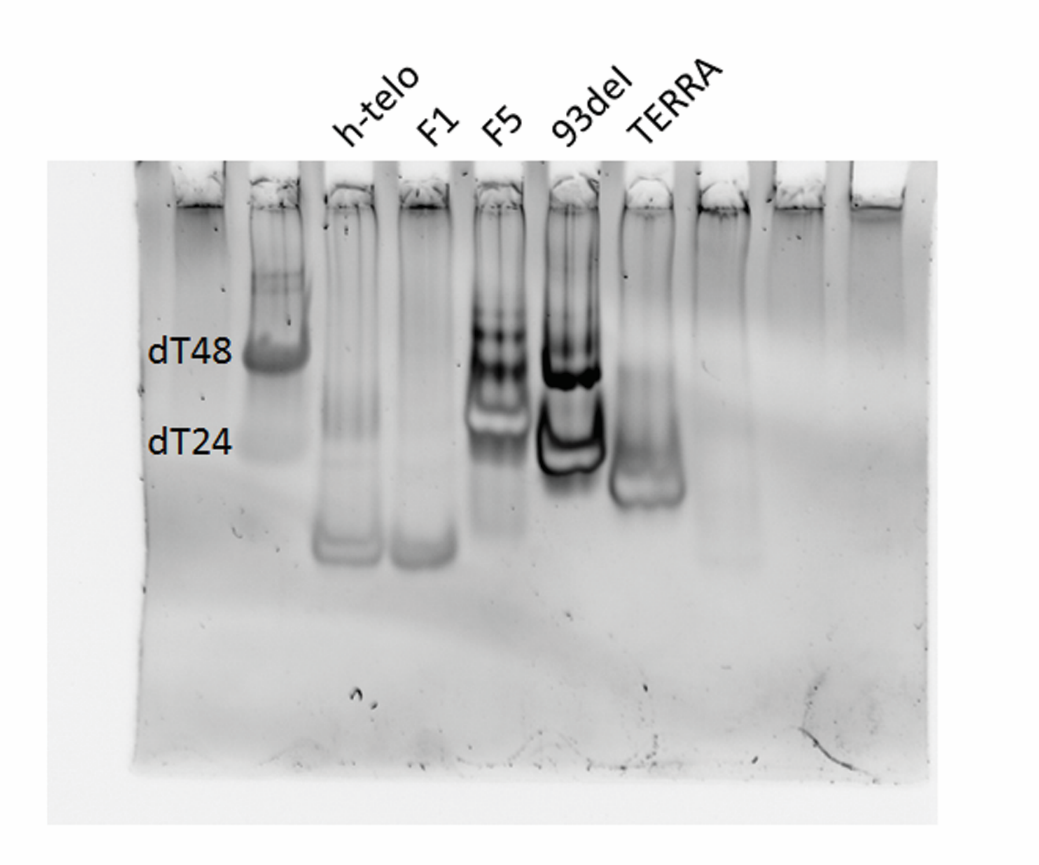


**Figure S2.** **Gel electrophoresis and staining of d(G4C2)_2_ fractions F1 and F5 in parallel with size indicators.** This is the original full-length gel figure. The F1 and F5 fractions were compared with monomeric human telomere h-telo (d[TAGGG(TTAGGG)_3_])[^1^](#_ENREF_1), dimeric G-quadruplex DNA 93del (d[GGGGTGGGAGGAGGGT])[^2^](#_ENREF_2) and dimeric human TERRA (r[UAGGGUUAGGGU])[^3^](#_ENREF_3) for electrophoretic mobilities. All the samples were prepared in non-denaturing 18% PAGE at 100 μM concentration. F1 migrated similarly as h-telo, supporting the dimeric form of d(G4C2)_2_ in this fraction. F5 migrated slower than 93del, which agreed with the tetrameric composition of d(G4C2)_2_ in this fraction. The dimeric TERRA migrated slower than h-telo and F1, suggesting different migration characteristics of DNA and RNA.


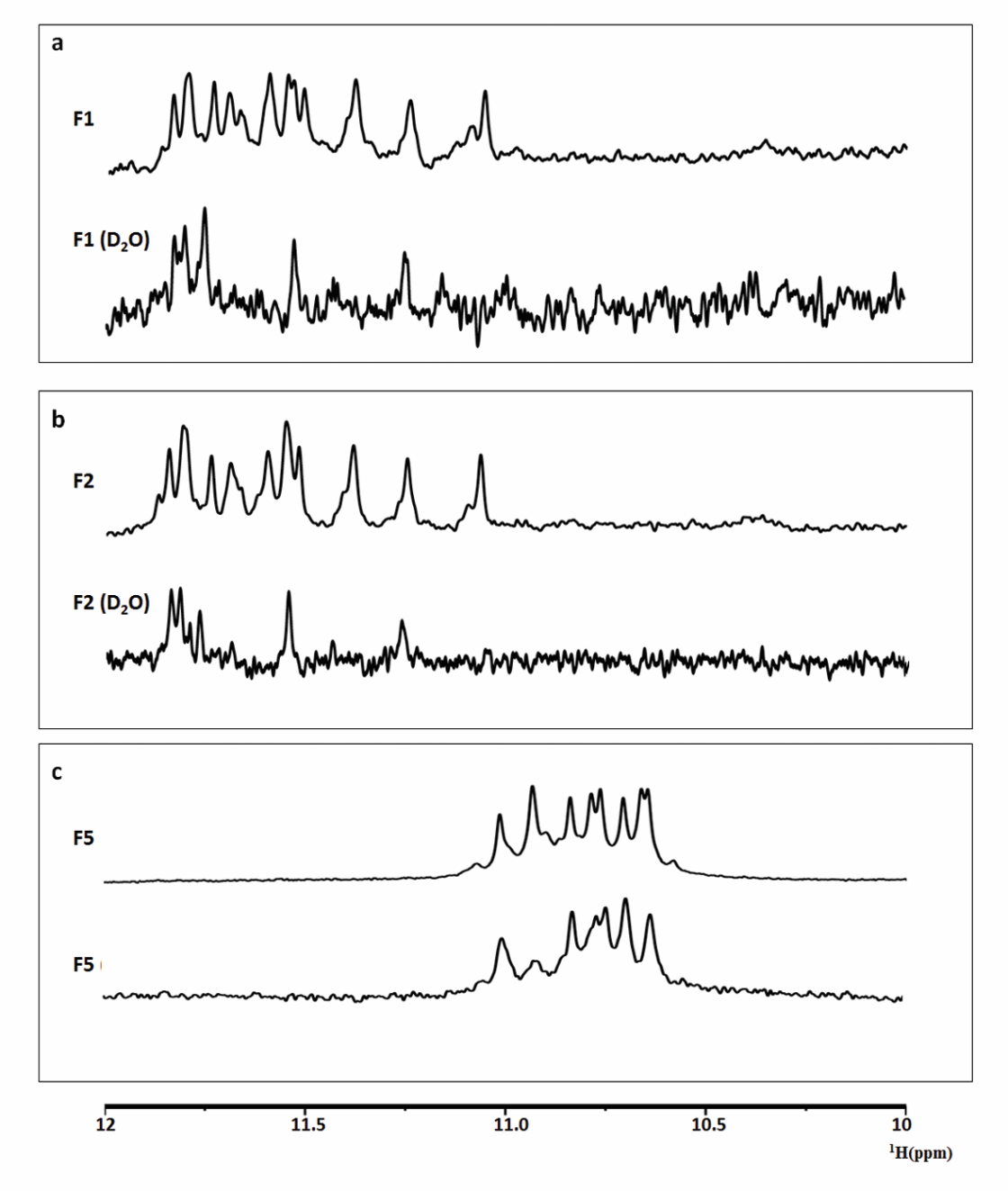


**Figure S3. HDX experiments of d(G4C2)_2_ fractions F1 (a), F2 (b) and F5 (c).** In each subfigure the top shows the imino region of the reference spectrum in 90% H_2_O, 10% D_2_O at 25 °C, while the bottom shows the imino proton spectrum after 30 min in 99% D_2_O.





**Figure S4. Crystallization of the d(G4C2)_2_ fraction F5.** (a) The crystals formed by the d(G4C2)_2_ fraction F5. (b) The diffraction image of a d(G4C2)_2_ F5 crystal.

**References**

1 Phan, A. T., Kuryavyi, V., Luu, K. N. & Patel, D. J. Structure of two intramolecular G-quadruplexes formed by natural human telomere sequences in K+ solution. *Nucleic Acids Res.* **35**, 6517-6525, (2007).

2 Phan, A. T. *et al.* An interlocked dimeric parallel-stranded DNA quadruplex: a potent inhibitor of HIV-1 integrase. *Proc. Natl. Acad. Sc.i U. S. A.* **102**, 634-639, (2005).

3 Martadinata, H. & Phan, A. T. Structure of propeller-type parallel-stranded RNA G-quadruplexes, formed by human telomeric RNA sequences in K+ solution. *J. Am. Chem. Soc.* **131**, 2570-2578, (2009).
